# Towards Healthy Aging: Influence of the Built Environment on Elderly Pedestrian Safety at the Micro-Level

**DOI:** 10.3390/ijerph18189534

**Published:** 2021-09-10

**Authors:** Muhan Lv, Ningcheng Wang, Shenjun Yao, Jianping Wu, Lei Fang

**Affiliations:** 1 Key Laboratory of Geographic Information Science (Ministry of Education), East China Normal University, Shanghai 200241, China; mhlv@stu.ecnu.edu.cn (M.L.); ncwang@stu.ecnu.edu.cn (N.W.); jpwu@geo.ecnu.edu.cn (J.W.); 2 School of Geographic Sciences, East China Normal University, Shanghai 200241, China; 3 Research Center for China Administrative Division, East China Normal University, Shanghai 200241, China; 4 Department of Environmental Science and Engineering, Fudan University, Shanghai 200438, China; fanglei@fudan.edu.cn

**Keywords:** elderly, pedestrian collisions, GWPR, built environment, walk, street view, safety, health

## Abstract

As vulnerable road users, elderly pedestrians are more likely to be injured in road crashes due to declining physical and perceptual capabilities. Most previous studies on the influence of the built environment on elderly pedestrian safety focused on intersections or areal units. Using a district of Shanghai as the study area, this research investigated the effects of the built environment at the road segment level with elderly pedestrian collision, taxi tracking point, point of interest, street view image, open street map, land use, housing price, and elderly population datasets. In particular, this research employed both Poisson and geographically weighted Poisson regression (GWPR) models to account for spatial nonstationarity. The Poisson model indicates that green space, sidewalks, and junctions on the roads significantly affected elderly pedestrian safety, and roads around nursing homes, schools, bus stops, metro stations, traditional markets, and supermarkets were hazardous for elderly pedestrians. The results of the GWPR model suggest that the influence of factors varied across the study area. Green space could decrease the risk of elderly pedestrian collisions only in areas without congested environments. Separations need to be installed between roadways and sidewalks to improve elderly road safety.

## 1. Introduction

The United Nations reported that, for the first time in history, the number of people aged 65 years or over exceeded the number of people under five in the world in 2018 [1]. At present, most developed nations and many developing countries are facing an aging problem. In China, the seventh population census reported that the aging population (people aged 60 years and older) accounted for 18.70% of the total population [2].

If older people can remain mobile and care for themselves longer, the costs for long-term care will be lower [3]. Walking, which is beneficial for both the physical and mental health of older people, has been widely acknowledged as the best exercise for the elderly [4]. However, as one of the most vulnerable road user groups, the elderly are more likely to be injured in road crashes when walking on streets due mainly to their declining physical and perceptual capabilities. For instance, lower mobility and delayed response times to approaching vehicles increase the chance of becoming a victim [5,6]. It is difficult for the elderly with reduced perceptual and cognitive capabilities to maintain general attention and distinguish between important and unimportant information, resulting in inaccurate, delayed, and inappropriate judgments in complicated situations [7,8,9]. Therefore, it is urgent to improve elderly pedestrian safety facing the challenge of rapid aging.

Environmental characteristics can influence the safety of elderly pedestrians despite physical limitations [10,11]. In the literature, only a few studies focused on the relationship between the built environment and elderly pedestrian safety. While early research usually concentrated on roadway characteristics such as intersection types, traffic volumes (traffic exposure), width, lanes, traffic signs, and crossings [12,13,14], recent studies have begun investigating community-level environmental features. Lam et al. [10] examined the influences of both roadway and community-level features on elderly pedestrian collisions in the urban area of Hong Kong. They found that elderly pedestrian collisions were more likely to happen on crossings and locations around bus stops and mixed land use. A recent study by Lee et al. [11] investigated the effects of facilities on elderly pedestrian safety by severity level in South Korea. They found significant determinants of BRT stations, department stores, banks, and traditional markets. Another study by Kim [15] reported that intersections with more parking lots, bus stations, commercial and office lands in the vicinity were likely to have a more significant number of elderly pedestrian collisions. They also found that intersections in residential, park, and recreational areas had a safer walking environment for older people. However, most previous studies on elderly pedestrian safety were conducted based on intersections [15,16] or areal units such as census tracks [17,18,19] (that is, elderly pedestrian collisions were aggregated by intersection or areal unit), limited research investigated middle sections of roads where the severity of traffic collisions is high. The only exception, to the best of the authors’ knowledge, was Lam et al. [10]. They aggregated elderly pedestrian crashes by road segment and analyzed the influence of the built environment at the road segment level. However, their research focus was to measure the elderly pedestrian exposure and examine the influence of the exposure variable. The built environment factors only included environmental variables on crossings, overpasses (underpasses), bus stops, and degree of land use mix. The impacts of the built environment have not been fully explored.

This study investigates the effects of the built environment on elderly pedestrian collisions at the road segment level in an urban area. In particular, this research introduces some facilities relevant to the activities of the elderly and some roadway features derived from street view images that may reflect perceptions of the elderly when they are walking along the streets. Additionally, this research employed both Poisson and geographically weighted Poisson regression (GWPR) to account for spatial nonstationarity.

## 2. Study Area and Data

### 2.1. Study Area

**Figure 1:**
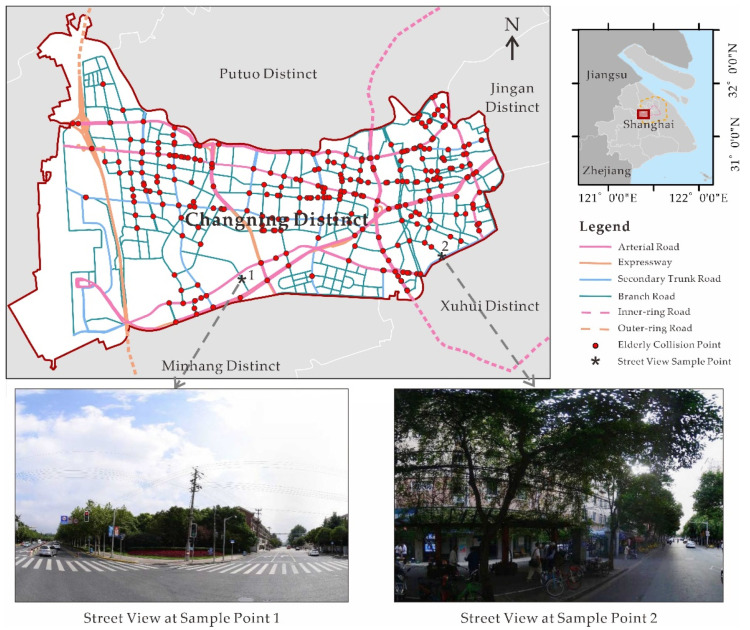
Location of the study area.

Located in the eastern part of China, Shanghai is one of the largest metropolitan cities in the world and has been experiencing a surge in the number and proportion of the elderly. According to China’s Population Census 2020, 23.4% of the residents were aged 60 or above in Shanghai [20]. It is worthwhile exploring elderly pedestrian safety in the city. There are 16 districts in Shanghai. As Changning District is located in the city’s downtown area with various road types, including expressways, and arterial, secondary trunk, and branch roads, we chose this district as the study area (see Figure 1). Figure 1 also describes the location of the Inner-ring and Outer-ring Roads. The area within the Inner-ring Road is the urban core, and that outside of the Outer-ring Road is the suburb.

### 2.2. Data

In this research, factors that may influence the occurrence of elderly pedestrian collisions can be classified into two categories. One type is known as exposure variables in road safety research [21]. Previous studies indicated that traffic flow or pedestrian volume positively affects the number of elderly pedestrian collisions [22,23], but the effects are usually nonlinear. The other type refers to built environment factors that can be further classified as roadway and community environment variables. The former describes the characteristics of the built environment on the roads such as crossings, traffic lights, sidewalks, and roadside trees that may influence the behavior of the elderly while walking, and the latter is used to delineate features of the community such as bus stops, supermarkets, shopping centers, and clinics that probably affect the daily activities of older people. Despite the limited research on the influence of the built environment on older pedestrians, many studies have focused on the effects of environmental factors on pedestrians of all ages [24,25,26,27]. This research selected variables by referring to previous studies on pedestrians regardless of age.

#### 2.2.1. Elderly Pedestrian Collision and Road Network Data

The elderly pedestrian collision data were collected from the Shanghai 110 Calling Center. A total of 3653 traffic collisions occurred in Changning District from 2013 to 2015, of which 410 involved elderly pedestrians. The database records the geographic coordinates and the address of each crash. As collisions are rare events, we aggregated the data from three years to ensure the representativeness of the sample. The road network dataset was derived from Open Street Map (OSM) [28]. By extracting locational information of collisions, the elderly pedestrian collisions were assigned to appropriate roads. Expressways were excluded in our study because only vehicles are allowed to pass through the type of road.

#### 2.2.2. Exposure Data

Exposure variables play a crucial role in crash prediction models [21]. In modeling vehicle–pedestrian collisions involving the elderly, vehicle and elderly pedestrian exposure variables are essential. As detailed traffic flow and elderly pedestrian volume were not available, this study employed taxi flow and the elderly population as proxies for exposure variables. Taxi GPS tracking points were collected by Shanghai Qiangsheng Taxi Company in April 2015. The database recorded detailed information on the driving condition of the vehicle, such as time, location (longitude and latitude), instantaneous speed, and whether the vehicle was carrying passengers. As taxies without passengers may have significantly different behavior with other vehicles, this research only included taxies with passengers. Elderly population data were provided by Shanghai Municipal Public Security Bureau in 2016. The dataset reported the number of elderly residents at each building.

#### 2.2.3. Built Environment Data

Street view images were used to portray characteristics of the road environment. They cannot only describe the road conditions but also, to some extent, reflect the visual perception of streetscapes [29,30]. We used Baidu static map API [31] to obtain street view images. The API allows developers to obtain images of streets by setting a set of parameters, such as image size, location, and direction of cameras. In this paper, street view sampling points were determined with about 100 m intervals, resulting in 810, 378, and 1455 points on the arterial, secondary trunk, and branch roads, respectively. Since the height of the panorama was four times the width, the image was set to 512 pixels × 1024 pixels (half the size of a panorama) with a vertical angle equal to zero degrees. The images of two headings (90 and 270 degrees) in the same location can form a panorama. Pyramid Scene Analysis Network (PSPNet) [32], one of the advanced deep learning methods for image semantic segmentation, was employed to derive features from street view images. We used the ADE20k scene parsing dataset to train the network and extract characteristics about the sky, vegetation, buildings, sidewalk, and streetlights [33].

Point of interest (POI) and land use data can, to some extent, reflect people’s activities, which may influence traffic conditions and the behavior of road users. The former were collected from the 2014 Baidu map API [34] and were classified into several groups such as bus stops, groceries, hospitals, clinics, restaurants, and schools. The latter were obtained based on the interpretation of aerial remote sensing images of the Shanghai Institute of Surveying and Mapping in 2015.

Housing price can be used to describe the socioeconomic characteristics of a community. We collected the housing price information in 2016 from FangTianXia [35], a real estate renting and selling service platform.

## 3. Method

Each road was divided into several segments with an equal interval. As elderly pedestrian collisions are rare events, a road segment has to be long enough to allow the significant variation of collision counts but short enough to reflect changes in the road environment [36]. Following the state-of-the-art network segmentation method, the road network was segmented at intervals of two hundred meters [37]. The elderly pedestrian crashes were aggregated by road segment, and the number of collisions happening on segments was used as the dependent variable [37]. Next, independent variables were derived from various sources of data. We used the collinearity test and calculated the variance inflation factor (VIF) value to select the variables for analysis. Then, the Poisson regression was chosen to model the influencing factors of elderly pedestrian crashes. Taking spatial nonstationarity into consideration, we used the geographically weighted Poisson regression (GWPR) model to analyze elderly pedestrian collisions.

### 3.1. Variable Selection

In this study, 18 independent variables related to exposure and built environment were identified for analysis. Table 1 presents descriptive statistics for the independent variables; all the VIF values were less than ten (ranging from 1.09 to 3.60), indicating small collinearity [38,39].

### 3.2. Global Collision Prediction Model—Poisson Regression

Many studies have suggested that generalized linear models, such as Poisson and negative binomial regression (NBR) model, perform higher accuracy in random and discrete data fitting [40,41,42]. Usually, NBR models will be employed instead of Poisson models if the data has an overdispersion problem. In this research, the overdispersion of data was not significant. Additionally, we tested the two kinds of models, and the values of Akaike information criterion (AIC) and Bayesian information criterion (BIC) were similar. Since we used the GWPR model that relies on Poisson distribution for dealing with nonstationarity, we finally decided to employ the Poisson model for consistency. The Poisson regression is as follows:

(1) $$ln\left(y_{i}\right)=\beta_{0}+\sum_{k}\beta_{k}x_{k,i}$$

where $y_{i}$ is the expected number of elderly pedestrian collisions on each road segment; $\beta_{0}$ is the intercept term, and $\beta_{k}$ represents the parameters to be estimated. Maximum likelihood was used to approximate the set of parameters ($\beta_{k}$).

### 3.3. Local Collision Prediction Model—Geographically Weighted Poisson Regression

As global collision prediction models such as Poisson regression are limited in capturing spatial heterogeneity in the collision data, this research also employed an advanced modeling technique, GWPR, to deal with spatial nonstationarity for frequency data [17,43,44,45,46]. The algorithm of GWPR is as follows:

(2) $$ln\left(y_{i}\right)=\sum_{k}\beta_{k}\left(X_{i},Y_{i}\right)x_{k,i}+\beta_{0}$$

where $y_{i}$ is the expected number of elderly pedestrian collisions in ith road segment; $\beta_{k}$ is the estimated coefficient of kth variable, $\beta_{0}$ is the intercept term; $\left(X_{i},Y_{i}\right)$ are the coordinates of the centroid of ith road segment, and $x_{k,i}$ represents the kth variable name in ith road segment.

Fotheringham et al. [45] used weighted least squares to estimate parameters by assuming that observed data closer to location *i* have a greater influence than those far away. It can indicate the degree of influence of different spatial locations on the estimation of regression point parameters. There are two commonly-used weight functions, Gaussian and Bi-square:

Gaussian:

(3) $$W_{ij}=\exp\left(-{\left(\frac{d_{ij}}{h}\right)}^{2}\right)$$

Bi-square:

(4) $$W_{ij}=\{\begin{array}{c} {\left[1-{\left(\frac{d_{ij}}{h}\right)}^{2}\right]}^{2},\;\;if\;d_{ij}<h_{i}\\ 0,\;\;otherwise \end{array}$$

Compared to the choice of weighted function, GWPR is more sensitive to the bandwidth. Due to the unbalanced distribution of elderly pedestrian collisions in Changning District, this research utilized Adaptive Bi-square, Golden section search, and the corrected Akaike information criterion to acquire the optimal bandwidth value. GWR4 software was employed to establish the GWPR model [47].

### 3.4. Measures of Goodness of Fit

Mean absolute deviation (MAD), mean squared prediction error (MSPE), and normalized root mean square error (NRMSE) were employed to measure the goodness of fit of the two models. Among these indicators, MAD represents the average misprediction of the model, MSPE indicates the model’s error associated with a prediction, and NRMSE summarizes the residual variance. For all of the three indicators, a lower value means better model performance. The algorithms are as follows:

(5) $$MAD=\frac{\sum_{i=1}^{N}\left|\hat{Y_{i}}-Y_{i}\right|}{N}$$

(6) $$MSPE=\frac{\sum_{i=1}^{N}{\left(\hat{Y_{i}}-Y_{i}\right)}^{2}}{N}$$

(7) $$NRMSE=\frac{\sqrt{\frac{\sum_{i=1}^{N}{\left(\hat{Y_{i}}-Y_{i}\right)}^{2}}{N}}}{Y_{max}-Y_{min}}$$

where *N* is the number of total road segments, $Y_{i}$ is the observed number of elderly pedestrian collisions on the ith road segment, $\hat{Y_{i}}$ is the predicted number of elderly pedestrian collisions on the ith road segment, and $Y_{max}$ and $Y_{min}$ are the maximum and minimum values of Y respectively.

### 3.5. Measure of Spatial Nonstationarity—Moran’s I

The degree of spatial autocorrelation of regression model residuals can help detect possible problems in spatial data modeling [43,48]. This study used Moran’s *I* statistic to test the spatial autocorrelation of residuals. The measure is given by [49,50]:

(8) $$I=\frac{n}{S_{0}}\;\frac{\sum_{i=1}^{n}\sum_{j=1}^{n}w_{ij}z_{i}z_{j}}{\sum_{i=1}^{n}z_{i}^{2}}$$

where $z_{i}$ is the deviation of residual for segment *i* from its mean, $w_{ij}$ is the spatial weight between road segment *i* and segment *j*, *n* is equal to the number of road segments, and $S_{0}$ is the aggregate of all the spatial weights:

(9) $$S_{0}=\overset{n}{\underset{i=1}{\sum }}\overset{n}{\underset{j=1}{\sum }}w_{ij}$$

The null hypothesis of Moran’s *I* is that the residuals are randomly distributed in the study area. The value falls between −1 and 1. A statistically significant *p*-value indicates that the residuals of the model are not randomly distributed.

## 4. Results and Discussion

### 4.1. Global Model–Poisson Regression

Under the assumption that the study area was homogeneous and there was no spatial relationship between road segments in the region, a global model was used to explore the relationship between dependent and independent variables. Table 2 presents the results of parameter estimates in the Poisson model. The two exposure variables, namely vehicle kilometers traveled by taxies (vehicle_km) and the number of elderly residents living nearby (num_elder), significantly and positively affected the occurrence of elderly pedestrian road crashes, with p-values less than 0.01. The result is consistent with previous findings that exposure variables are the most important predictors of road crashes regardless of the road collision type [21,51].

For roadway variables, the number of junctions (num_junction) had significant impacts on pedestrian road crashes. The result provides additional evidence that road junctions are dangerous places for the elderly because of the complex traffic environment and the physical limitations of older people [10,15]. Regarding the features derived from the street view images, the average proportion of green space (p_green) or sidewalk space (p_sidewalk) was a significant predictor. The coefficient of the p_green supports that green space can positively influence the health of older people by reducing traffic injury. The reason could be that the green space might affect the behavior of elderly pedestrians [52]. The sign of the p_sidewalk coefficient was positive, indicating that sidewalks may not necessarily protect the elderly and may even increase their risks of being victims in road collisions, in accordance with Lam et al. [10]. A possible reason might be that no separation is installed between sidewalks and roadways in some places (see the street view image at sample point 2 in Figure 1), particularly on lower-order roads where there is a complex traffic environment. People may walk from sidewalks to roadways easily. They probably walk across roads anytime for convenience, which could significantly increase the risk of colliding, especially for older people with a lower response capability [10]. Additionally, if a driver sees more sidewalk space in their view, they could probably believe that the travel environment is relatively safe because pedestrians would have enough space for walking. This might result in the incautiousness of the driver, which may increase the chance of colliding with elderly pedestrians when they cross the roads.

Communities with more schools may have higher risks for elderly pedestrians (see variable of num_school). In China, especially in metropolitan cities such as Shanghai, seniors and the elderly are usually responsible for taking care of their grandchildren in the daytime. They always take their grandchildren to and back from school on foot. The travel environment is highly complex around schools in the morning when children go to school and in the afternoon when classes are over, owing to the traffic mix, including cars, bicycles, and electric motorcycles with highly concentrated road users. This may pose a significant threat for the elderly. Consistent with previous studies such as Lee et al. [11] and Kim [15], locations around bus stops, metro stations, traditional markets, and supermarkets are also dangerous for older people (see variables of num_station and num_market in Table 2), due probably to the unfriendly walking environment for people with physical limitations. For instance, there are always many dockless shared bicycles parking around these places and sometimes in a disorderly manner. Nursing homes are crucial for an aging society. The result of this study suggests that locations around nursing homes might be hazardous. For one thing, older people might visit their friends who live in the nursing homes; for another, people would visit the nursing home to better know the living condition before applying for admission. These activities would increase the elderly pedestrian flow, resulting in the growth of elderly pedestrian collision counts. Similar to the findings of Kim [15], roads near residential areas might have more significant risks for older people (see the variable of area_residential), probably because of the complicated traffic around estates. The variable num_medical negatively influenced the occurrence of elderly pedestrian collisions at the significance level of 90%. It suggests that locations around hospitals and clinics might be safe, probably thanks to appropriate commands by guards nearby. The action could be more helpful for vulnerable road users.

### 4.2. Local Model—Geographically Weighted Poisson Regression

The GWPR model was established to account for spatial nonstationarity. We calculated the Moran’s *I* statistics of the residuals of predictions using the Poisson and GWPR models. For the former, there was a positive spatial autocorrelation in the residuals at a 95% level of significance, reflecting that the regression coefficients of the global model could not adequately represent detailed local variations in the data and the residuals were spatially correlated with clustering tendency. For the latter, the spatial autocorrelation of the residuals was not statistically significant at the 95% level. Given the same variables, the local model was better than the global model in accounting for the spatial nonstationarity of the elderly pedestrian collision data at the road segment level in the study area. Table 3 illustrates the overall performance of the two models. The result indicates that GWPR could better model elderly pedestrian road crashes than the Poisson model.

Table 4 presents descriptive statistics of the coefficients of GWPR. The values of coefficients vared significantly across the study area regardless of the variable. We also calculated significance levels by variable and road segment. The result indicated that the impact of a variable on elderly pedestrian safety might not be necessarily significant on every local segment, even it was found significant in a global model. We selected factors that influenced elderly pedestrian collision risks at the 95% significance level or above in the Poisson model for further analysis. These variables had relatively more significant units than others.

**Figure 2:**
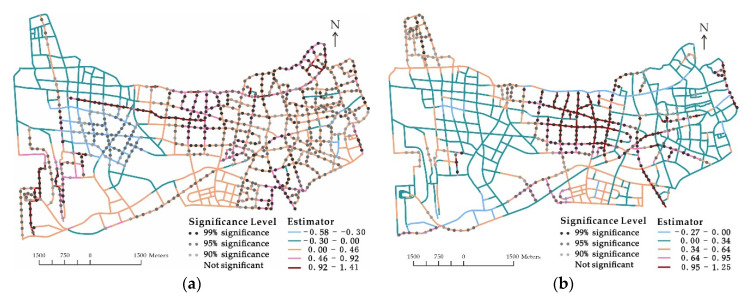
Spatial distribution of coefficient estimations and significance levels by exposure variable. (**a**) vehicle_km and (**b**) num_elder.

Figure 2 delineates the spatial distribution of coefficient values and significant levels of the GWPR model by exposure variable. Although the two variables significantly and positively influenced the number of elderly pedestrian road crashes with *p*-values less than 0.01 in the global model, they were detected as not significant on some of the local road segments in the GWPR model. The problem was severe for the variable of num_elder. The reason could be that we used the number of elderly residents at a community level rather than the elderly pedestrian flow at a road segment level to represent the elderly pedestrian exposure because of data unavailability. The former could not capture specific types of older persons’ activities such as purchasing food in traditional markets, visiting friends at nursing homes, or taking grandchildren to school. Therefore, the variable could not fully reflect the activities of the elderly at a segment level [21]. Nonetheless, in the absence of detailed elderly pedestrian volume at each road segment, it was suitable to introduce a proxy such as the number of elderly residents to reflect the variation of elderly pedestrian flow.

**Figure 3:**
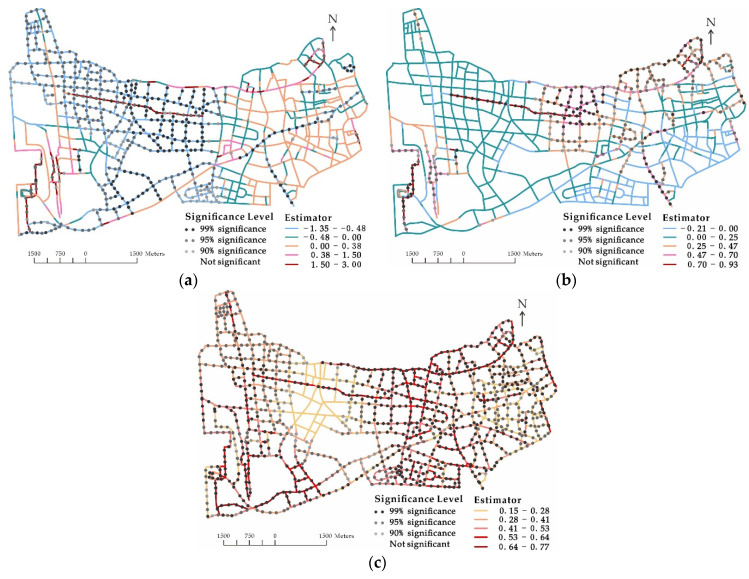
Spatial distribution of coefficient estimations and significance levels of by roadway variable. (**a**) p_green, (**b**) p_sidewalk, and (**c**) p_numjunction.

The coefficient values and significant levels of roadway variables are portrayed in Figure 3. Interestingly, most road segments with significant p_green are located far from the center (Figure 1 and Figure 3a). This suggests that green plants could not improve elderly pedestrian safety in the congested built environment, due probably to their possible negative influence on the vision of older road users. Looking closely at types of road segments with significant p_sidewalk (Figure 1 and Figure 3b), one may identify that the most significant units were lower-order roads where there were complicated travel environments with no separations installed between roadways and sidewalks. The spatial distribution of road segments with significant num_junction (see Figure 3c) once again provided strong evidence that road junctions were hazardous for elderly pedestrians.

**Figure 4:**
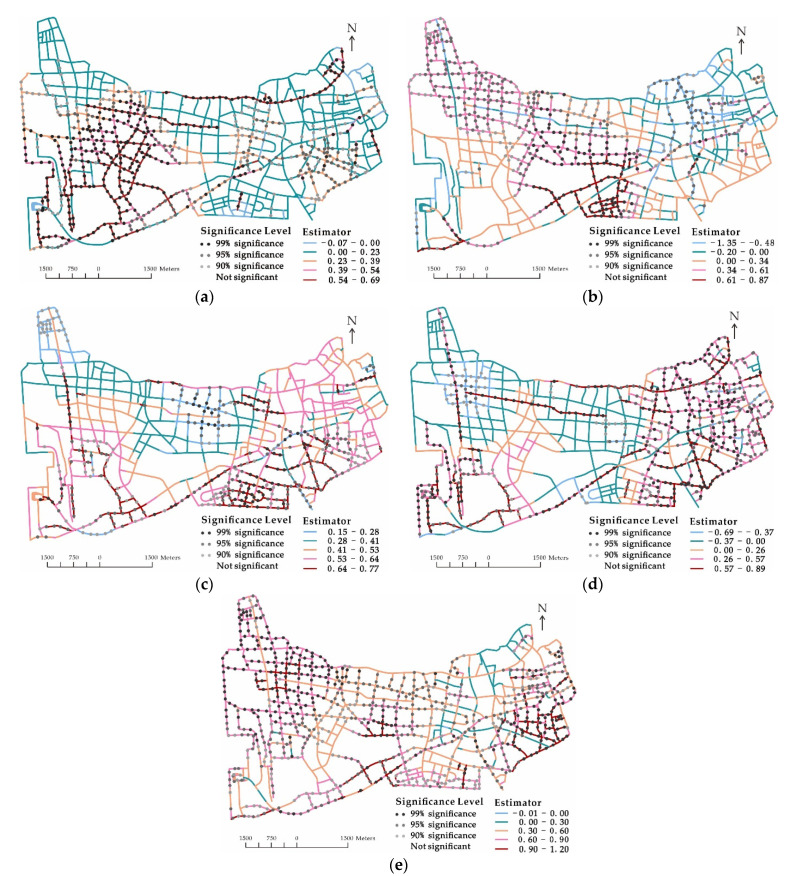
Spatial distribution of coefficient estimations and significance levels of by community variable. (**a**) num_nursing, (**b**) num_school, (**c**) num_station, (**d**) num_market, and (**e**) area_residential.

Figure 4 describes the spatial variation of coefficient values and significant levels by community variable. While most road segments with significant num_nursing or num_school were distributed far from the city center, the spatial units with significant num_market were concentrated in the core area. Regardless of the location, the variable area_residential had positive and significant impacts on elderly pedestrian road crashes at almost every road segment. It highly suggests that the road environment was not walkable for the elderly in residential areas. The results show that we should take countermeasures on specific places in different regions. For instance, more traffic facilities can be installed around nursing homes near the Outer-ring Road, and safeguards can be assigned to help direct traffic at the gate of traditional markets in the urban core area.

## 5. Conclusions

The World Health Organization defines healthy aging as “the process of developing and maintaining the functional ability that enables wellbeing in older age” [14]. Walking can help maintain the functional ability of the elderly. As the built environment is highly influential on our behavior, a walkable environment for the elderly is desirable towards healthy aging. In this research, the effects of the built environment on elderly pedestrian collisions were investigated at the road segment level in an urban area with both Poisson and GWPR models. The result indicated that roads around nursing homes, schools, bus stops, metro stations, traditional markets, and supermarkets were hazardous for elderly pedestrians. The elderly were more likely to be involved in a traffic collision on road junctions and middle segments near residential areas. It was also found that the influence of factors varied across the study area. Green space could decrease the risk of elderly pedestrian collisions only in areas without congested environments. Transportation planners and engineers should work together to improve elderly road safety. For instance, elderly-friendly crossing facilities such as pedestrian refuges could be built in the middle of roads because the elderly walk at a relatively low speed. The refuges could help them cross roads safely. Separations need to be installed between roadways and sidewalks, notably on lower-order roads such as branch roads. Moreover, close collaboration among a wide range of stakeholders is necessary for improving elderly pedestrian safety. A typical example is exploring the influence of green space on the walking behavior of the elderly. It may require joint efforts from multidisciplinary specialists such as psychologists, geographers, and urban planners.

One limitation of this research was the exposure variable. As the detailed elderly pedestrian flow was unavailable, this research used the number of elderly residents living around the road segments as a proxy. As the exposure variable can help better understand the influence of risk factors, more research efforts can be dedicated to measuring the elderly pedestrian flow at the micro-level. One more limitation was that we aggregated crashes within 24 h without considering temporal variation because of the sample size. However, a more indepth analysis of elderly pedestrian collisions by the time of day will shed great light on the influence of the glare and brightness due to the afternoon or morning orientation of the sun. For instance, when the sun is lower in the sky, it may be difficult for both the driver and the pedestrian to see each other, resulting in a greater risk of colliding. Additionally, we found that green space could improve the safety of elderly pedestrians. However, the way in which greenness influences the traveling behavior of the elderly remains unclear. As a further step, more experiments should be conducted to illustrate the mechanism.

## Figures and Tables

**Table 1:** Descriptive statistics of dependent and independent variables.

Variable	Description	Max	Min	Avg	SD	VIF
Dependent Variable						
elder_collision	No. of elderly pedestrian collisions	7	0	0.333	0.8076	-
Exposure Variable						
vehicle_km	Vehicle kilometers travelled by taxies	1,220,000	0	120,000	153,000	2.072
num_elder	No. of persons aged 60 or above within 500 m buffer	63.577	0.1	23.953	12.859	2.271
Roadway Variable						
p_green	Average proportion of green space	0.690	0	0.230	0.143	1.815
p_sky	Average proportion of sky space	0.638	0	0.342	0.130	1.686
p_building	Average proportion of building space	0.610	0	0.171	0.101	1.101
p_sidewalk	Average proportion of sidewalk space	0.112	0	0.029	0.018	1.631
speed_limit	Speed limit	60	40	44.602	7.602	1.643
num_junction	No. of road junctions	3	0	0.762	0.639	2.792
rd_width	Road width	21	3	6.896	2.676	1.153
Community Variable						
num_nursing	No. of nursing homes within 500 m buffer	4	0	0.663	0.879	1.258
num_school	No. of schools within 500 m buffer	10	0	2.441	2.383	1.402
num_station	No. of bus stops and metro stations within 500 m buffer	79	0	28.621	16.080	3.600
num_medical	No. of hospitals and clinics within 500 m buffer	10	0	2.074	1.851	3.129
num_market	No. of traditional markets and supermarkets within 500 m buffer	34	0	7.416	7.030	2.487
num_park	No. of parks and squares within 500 m buffer	4	0	0.579	0.848	1.254
area_green	Area of green land (sq. m)	198,000	0	29,031	28,423	1.087
area_residential	Area of residential land (sq. m)	755,000	0	395,000	194,000	2.438
area_commercial	Area of commerical land (sq. m)	326,000	0	24,851	50,169	2.035
avg_price	Average house price (RMB Yuan) within 500 m buffer	165,210	0	42,731	17,245	1.743

SD, standard deviation; VIF, variance inflation factor.

**Table 2:** The estimation results of the global Poisson model for independent variables.

Variable	Coef.	Robust Std. Err.	*z*	*p* > |*z*|	95% Confidence Interval	
vehicle_km	0.173	0.064	2.720	0.007 ***	0.048	0.298
num_elder	0.309	0.086	3.580	<0.001 ***	0.140	0.478
p_green	−0.316	0.144	−2.200	0.028 **	−0.598	−0.035
p_sky	−0.014	0.127	−0.110	0.910	−0.263	0.235
p_building	−0.146	0.094	−1.560	0.118	−0.329	0.037
p_sidewalk	0.236	0.085	2.800	0.005 ***	0.071	0.402
speed_limit	0.064	0.079	0.810	0.416	−0.091	0.219
num_junction	0.380	0.057	6.670	0.000 ***	0.268	0.492
rd_width	0.088	0.065	1.350	0.177	−0.040	0.215
num_nursing	0.158	0.059	2.660	0.008 ***	0.041	0.274
num_school	0.152	0.077	1.970	0.049 **	0.001	0.302
num_station	0.155	0.068	2.270	0.023 **	0.021	0.289
num_medical	−0.136	0.081	−1.690	0.091 *	−0.294	0.022
num_market	0.164	0.079	2.060	0.039 **	0.008	0.319
num_park	0.001	0.070	0.020	0.987	−0.136	0.138
area_green	−0.134	0.094	−1.430	0.153	−0.317	0.050
area_residential	0.441	0.113	3.910	<0.001 ***	0.220	0.662
area_commercial	−0.034	0.071	−0.470	0.635	−0.174	0.106
avg_price	−0.122	0.132	−0.920	0.358	−0.381	0.138
_cons	−1.550	0.085	−18.250	<0.001 ***	−1.717	−1.384
AIC: 1749.306; BIC: 1851.634						

* Significant at 0.1 level; ** Significant at 0.05 level; *** Significant at 0.01 level. Coef., coefficient; Std. Err., standard error; AIC, Akaike information criterion; BIC, Bayesian information criterion.

**Table 3:** Model overall performance of two models.

Model	MAD	MSPE	NRMSE
Poisson	0.536	0.761	0.383
GWPR	0.366	0.395	0.170

GWPR, geographically weighted Poisson regression; MAD, mean absolute deviation; MSPE, mean squared prediction error; NRMSE, normalized root mean square error.

**Table 4:** Descriptive statistics of coefficients of GWPR.

Variable	Min	Max	Lower Quartile	Median	Upper Quartile
vehicle_km	−0.588	1.400	−0.120	0.274	0.400
num_elder	−0.268	1.249	0.180	0.293	0.483
p_green	−1.348	2.992	−0.737	0.190	0.084
p_sky	−0.750	4.105	−0.290	0.072	0.206
p_building	−0.726	2.307	−0.279	−0.095	0.020
p_sidewalk	−0.203	0.925	0.019	0.117	0.316
speed_limit	−0.381	0.551	−0.111	0.025	0.169
num_junction	0.165	0.765	0.292	0.374	0.486
rd_width	−0.351	0.482	−0.115	−0.001	0.179
num_nursing	−0.076	0.693	0.124	0.211	0.410
num_school	−0.470	0.874	−0.078	0.185	0.409
num_station	−0.462	0.631	−0.073	0.182	0.314
num_medical	−0.804	0.451	−0.477	−0.094	0.104
num_market	−0.695	0.890	−0.213	0.219	0.465
num_park	−0.925	0.598	−0.213	−0.043	0.154
area_green	−0.859	0.564	−0.366	−0.135	0.054
area_residential	−0.006	1.176	0.480	0.611	0.775
area_commercial	−0.683	0.612	−0.136	−0.022	0.128
avg_price	−1.167	0.839	−0.451	−0.017	0.387
Intercept	−3.688	−1.433	−2.480	−1.885	−1.701
AIC: 1076.787; BIC: 1785.385					

## Data Availability

The data that support the findings of this study are available from the corresponding author, upon reasonable request.
